# Overview of Pregnancy in Renal Transplant Patients

**DOI:** 10.1155/2016/4539342

**Published:** 2016-11-30

**Authors:** Silvi Shah, Prasoon Verma

**Affiliations:** ^1^Department of Nephrology and Hypertension, University of Cincinnati, Cincinnati, OH, USA; ^2^Division of Neonatal/Perinatal Medicine, Brown University, Providence, RI, USA

## Abstract

Kidney transplantation offers best hope to women with end-stage renal disease who wish to become pregnant. Pregnancy in a kidney transplant recipient continues to remain challenging due to side effects of immunosuppressive medication, risk of deterioration of allograft function, risk of adverse maternal complications of preeclampsia and hypertension, and risk of adverse fetal outcomes of premature birth, low birth weight, and small for gestational age infants. The factors associated with poor pregnancy outcomes include presence of hypertension, serum creatinine greater than 1.4 mg/dL, and proteinuria. The recommended maintenance immunosuppression in pregnant women is calcineurin inhibitors (tacrolimus/cyclosporine), azathioprine, and low dose prednisone; and it is considered safe. Sirolimus and mycophenolate mofetil should be stopped 6 weeks prior to conception. The optimal time to conception continues to remain an area of contention. It is important that counseling for childbearing should start as early as prior to getting a kidney transplant and should be done at every clinic visit after transplant. Breast-feeding is not contraindicated and should not be discouraged. This review will help the physicians in medical optimization and counseling of renal transplant recipients of childbearing age.

## 1. Introduction

The first successful pregnancy in kidney transplant recipient occurred in 1958 to 23-year-old Edith Helm who received a kidney from her identical twin sister in 1956 and she delivered a healthy full-term boy of 3300 grams by cesarean section. Her twin sister, Wanda Foster, also gave birth four times successfully after donating the kidney [1]. Since then, there have been many successful pregnancies that have been reported in kidney transplant recipients offering hope to women who have always wished to conceive.

Current knowledge of outcomes of pregnancy in kidney transplant is limited from case reports, single-center studies, and four voluntary registries including National Transplantation Pregnancy Registry (NTPR) in the United States established in 1991 which is the only active registry, National Transplant Pregnancy Registry in the United Kingdom initiated in 1997, European Dialysis and Transplant Association Registry, and the Australian and New Zealand Dialysis and Transplant Registry. Small patient numbers and unavoidable reporting bias limit all these registries [2–5]. We have to keep in mind that most of our current knowledge that guides the management of pregnancy in renal transplant recipients comes from these retrospective studies.

## 2. Sexual Function

Women with chronic kidney disease (CKD) have abnormal hypothalamus-pituitary-ovarian axis that leads to menstrual cycle irregularity, anovulation, decreased libido, and impaired fertility. There is an earlier onset of menopause in women with CKD on an average by 4.5 years as compared to general population [6, 7]. Among women on hemodialysis, 73% have menstrual disorders and amenorrhea present in half of them [8]. Women with end-stage renal disease (ESRD), especially with amenorrhea, have high serum prolactin due to impaired renal clearance, increased luteinizing hormone (LH) and follicular stimulating hormone (FSH), and reduced estradiol and progesterone concentration. The persistently elevated gonadotropins due to loss of negative feedback on hypothalamic and pituitary centers and absence of LH surge lead to anovulation [6, 8, 9]. Pregnancy is therefore rare in women on dialysis with very low incidence of conception ranging from 0.9 to 7%. Even after conceiving successfully, the incidence of viable fetal outcome remains low at 20 to 40% [10].

However, the temporary change to hypogonadotropic hypogonadism takes place as soon as 2-3 weeks with return of circulating sex steroids to normal range within 6 months after successful renal transplantation [11]. Due to rapid restoration of hypothalamic-pituitary-gonadal axis, it becomes imperative that contraception should be started immediately after transplant in women with childbearing potential [12].

## 3. Effect of Pregnancy on Allograft Function

A normal pregnancy leads to hyperfiltration, intrarenal vasodilation, and increase in effective plasma flow with no concomitant increase in intraglomerular pressure. There is an increase in the glomerular filtration rate by about 50% with decrease in the serum concentration of creatinine and urea [13]. Renal allograft is able to adapt to physiological changes of pregnancy with an increase in creatinine clearance of approximately 30% in the first trimester which is sustained with a small decrease in the second trimester and returns to prepregnancy level during the third trimester [14]. Davison reported that the increase in 24-hour creatinine clearance in healthy women was comparable to allograft recipients at 10 weeks of gestation (38% versus 34%). Allograft recipients also have a higher 24-hour protein excretion as compared to healthy women which increases throughout pregnancy, becomes threefold higher by third trimester regularly exceeding 500 mg (versus 200 mg in healthy women), and returns to prepregnancy levels at 3 months postpartum [15]. Proteinuria in pregnancy should never be attributed to normal pregnancy related changes and common comorbidities like urinary tract infection and preeclampsia should be ruled out.

## 4. Risk of Maternal Complications

### 4.1. Hypertension and Preeclampsia

Hypertension is common in kidney transplant recipients with a reported incidence of 52% to 69%. The incidence of preeclampsia in renal transplant recipients ranges between 24% and 38% with a 6-fold higher risk compared to incidence of 4-5% in general population [4, 16–18]. It is difficult to distinguish preeclampsia from hypertension in renal transplant recipients because of the frequent increase in blood pressure after 20 weeks in previously normotensive women and hyperfiltration related worsening of preexisting proteinuria. Hyperuricemia becomes a less reliable marker for diagnosing preeclampsia since renal transplant recipients are usually on calcineurin inhibitors which also increase uric acid levels [19]. In addition, sudden worsening of hypertension and marked increase in the proteinuria are also noted in acute rejection, which further makes the diagnosis of preeclampsia challenging. Hypertension during pregnancy increases the risk of preterm delivery, intrauterine growth retardation, and the risk of graft loss [18].

Antihypertensives should be initiated if the blood pressure is consistently higher than 140/90 mmHg. Alpha-methyldopa and hydralazine are the traditional agents that have been used safely for controlling blood pressure during pregnancy. Other antihypertensives that are safe to be used in pregnancy include beta-blockers and calcium channel blockers. Angiotensinogen converting enzyme inhibitors are contraindicated due to their association with pulmonary hypoplasia and oligohydramnios in fetus. Low dose aspirin reduces the risk of preeclampsia in high-risk population and should be given to all renal transplant recipients [20].

### 4.2. Allograft Function

Pregnancy in the absence of risk factors does not increase the rate of graft loss. The graft failure rate did not differ in pregnant women as compared to nonpregnant allograft recipients at follow-up of 10 years (19% versus 21%) [14]. Risk factors associated with graft loss include history of drug treated hypertension, prepregnancy creatinine ≥ 1.4 mg/dL, and proteinuria. It was demonstrated in the NTPR registry that, out of 133 female renal transplant recipients, 20 who lost graft within 5 years had higher serum creatinine before pregnancy (1.6 mg/dL versus 1.1 mg/dL), higher serum creatinine after pregnancy (2.2 mg/dL versus 1.3 mg/dL), and higher incidence of rejection during or within 3 months after pregnancy (45% versus 4.6%). The risk of allograft loss at 5 years was 3.3-fold higher if prepregnancy creatinine was >1.3 mg/dL and 7.4-fold higher if prepregnancy creatinine was >1.6 mg/dL [4]. Keitel et al. reported that prepregnancy creatinine was >1.5 mg/dL in all six women who suffered graft loss within 2 years postpartum [21]. Sibanda et al. showed that there was no evidence of increased renal allograft loss after pregnancy in matched case control study but the 2-year postpregnancy graft survival was lower in allograft recipients with hypertension as compared to those without (100% versus 87%) [18]. The presence of nephrotic range proteinuria increases the risk of spontaneous abortion, intrauterine growth retardation, and prematurity in pregnant women and therefore it is recommended that proteinuria should be ≤500 mg before pregnancy in renal transplant recipients [22].

### 4.3. Risk of Rejection and Its Treatment

Pregnancy is a state of immunological tolerance associated with immunodepressant activity of lymphocytes which creates tolerance to fetus and may benefit the renal allograft; however, there is a possibility that the antigenic stimulus provided by the fetus may trigger graft rejection as well. In addition, acute rejection may be higher in the postpartum period due to return to normal immunosurveillance status [23]. The rate of allograft rejection is not increased during pregnancy or 3 months postpartum and varies between 1 and 14.5%, which is comparable to nonpregnant transplant recipients [4, 22]. Risk factors that increase the risk of rejection include high serum creatinine, rejection before pregnancy, and changing levels of immunosuppressive drugs but not the different immunosuppression regime [24]. The diagnosis of rejection is difficult, since rejection is frequently associated with a small rise in creatinine and could be confounded due to hyperfiltration related decrease in creatinine during pregnancy. It is safe to do ultrasound guided allograft biopsy during pregnancy to diagnose rejection [25]. High dose steroids have been successful in treating allograft rejection during pregnancy and remain first-line treatment. Data on the use of other agents like antithymocyte globulin and rituximab for treatment of rejection in pregnancy are limited and there are no specific recommendations [26].

### 4.4. Infections

Pregnant renal transplant recipients have a higher risk of infections, especially bacterial urinary tract infections (UTI) and acute pyelonephritis, due to use of immunosuppressive medications. UTI is present in up to 40% of women due to reflux, mild hydronephrosis after transplant, and pregnancy related dilation of renal collecting ducts and ureters. Screening for UTI should be performed with dipstick at every visit and with urine cultures at 4-week intervals. Asymptomatic bacteria should be treated with antibiotics for 2 weeks and then prophylaxis should be continued throughout pregnancy. Antibiotics used to treat UTI include nitrofurantoin and cephalexin [22]. Primary cytomegalovirus (CMV) infection results in 40–50% transmission to fetus with 5–18% of them being symptomatic at birth; however, secondary infection has a lower risk of affecting fetus (~2%). The diagnosis of fetal CMV is done by culture of the amniotic fluid. Congenital CMV is associated with hearing loss, learning problems, microcephaly, mental retardation, and perinatal death. The treatment of mother with ganciclovir or CMV hyperimmunoglobulin to prevent fetal CMV disease has not been demonstrated [27]. Herpes simplex infection in mother is associated with increased risk of abortion and can be transmitted from mother to the child during birth. Treatment is with acyclovir and caesarean section is preferred since it attenuates the risk of neonatal herpes. Infants born to mothers who carry hepatitis B antigen should be given hepatitis B immunoglobulin and hepatitis B vaccine to prevent neonatal infection, which offers protection for more than 90% of infants. Vertical transmission with hepatitis C remains low at <7%.

### 4.5. Other Obstetrical Complications

The risk of cesarean section in renal transplant recipients is higher than the general populations with a reported incidence of 43–64% [4, 16, 17]. Bramham et al. reported that the likelihood of cesarean section in renal transplant recipients in UK transplant registry was 5-fold higher and was twice as common as compared to general population (64% versus 24%), with majority being for fetal distress and 3% being performed merely due to the presence of renal allograft [17]. The incidence of gestational diabetes mellitus is not increased in pregnant women with renal transplants and ranges between 3% and 8% [16, 17].

## 5. Risk of Fetal Complications

The rate of live births in allograft recipients is comparable to general population and ranges from 71 to 79% [4, 18]. The incidence of preterm delivery has been reported to be as high as 40 to 60% versus 5 to 15% in general population and occurs mostly due to maternal or fetal compromise rather than spontaneous preterm labor [4]. High serum creatinine ≥1.7 mg/dL and presence of maternal hypertension predispose to preterm delivery [18]. In addition, they have high incidence of preterm birth (52 to 53%), low birth weight (42 to 46%), and IUGR (30 to 50%) [4, 23, 28]. Renal allograft recipients have a 13-fold higher risk of preterm deliveries, 12-fold higher risk of low birth weight babies, and 5-fold high risk of small for gestation babies as compared to general population as reported in a study by Bramham et al. [17]. The mean gestational age for newborn is 35.6 weeks with mean birth weight of 2420 grams [16]. The miscarriage rate ranges from 11 to 26% (versus 8 to 9% in general population) but there is no higher risk of perinatal mortality in the absence of risk factors of hypertension, proteinuria, and impaired allograft dysfunction [4, 17, 18].

## 6. Predictors of Pregnancy Outcomes

Risk factors described in association with poor pregnancy outcomes are hypertension, elevated prepregnancy creatinine ≥1.4 mg/dL, proteinuria, and history of ≥2 renal transplants. There was about 6-fold higher likelihood of poor fetal outcome (still birth, miscarriage, neonatal death, birth < 32 weeks, and congenital anomalies) in women with high prepregnancy creatinine and high diastolic pressure during second and third trimesters as reported in a study by Bramham et al. [17]. The presence of nephrotic range proteinuria increases the risk of spontaneous abortion, intrauterine growth retardation, and prematurity in pregnant women [22]. Therefore, prepregnancy creatinine ≤1.4 mg/dL, absence of hypertension, and minimal proteinuria <500 mg before pregnancy are associated with successful pregnancy outcomes. In addition, young age at pregnancy and young age at transplantation are associated with a higher likelihood of successful outcomes of live births. The duration of dialysis or history of living donation is not a predictor of successful pregnancy [14].

## 7. Optimal Time to Conception

The optimal time to conception after renal transplant continues to remain an area of contention. The ideal time of conception in women with renal transplant is between 1 and 2 years according to guidelines by American Society of Transplantation. European best practice guidelines recommend delaying pregnancy for a period of 2 years after transplantation [22, 29]. However, it is safe to conceive even after 6 months of getting a kidney transplant provided that the graft function is stable and that women are not on teratogenic medications. There is a higher likelihood of viable fetal outcome when conception is within 2 years of getting a transplant [16]. In addition, by that time, the viral prophylaxis has been completed and the immunosuppressive medication is at its nadir. Waiting longer may also result in impaired renal function postpartum which may fail to recover with already declining renal function due to chronic allograft nephropathy. However, a recent study reported that there is an increased risk of allograft failure to pregnancies in both first posttransplant year (HR: 1.25; 95% CI: 1.04, 1.50) and second posttransplant year (HR: 1.26; 95% CI: 1.06, 1.50), while pregnancy in the third posttransplant year was not associated with an increased risk of death censored graft loss [30].

## 8. Immunosuppression

Management of immunosuppression in pregnant renal transplant recipients is important due to the concern for teratogenic risk and potential adverse effects. All the immunosuppressive drugs cross the maternal-fetal circulation and have been detected in variable degrees in fetal circulation [31]. The Food and Drug Administration (FDA) categorizes drugs for pregnancy safety as follows: A (no human risk), B (animal studies showing risk but no evidence of human risk), C (human risk not ruled out), D (evidence of human risk), and X (absolutely contraindicated). The majority of the drugs fall into category C, where risk and benefits have to be weighed. The commonly used immunosuppressive drugs used in renal transplant recipients and their pregnancy information are summarized in Table 1 [32].

### 8.1. Calcineurin Inhibitors

Calcineurin inhibitors including tacrolimus and cyclosporine are considered safe during pregnancy. Calcineurin inhibitors cross the placenta and enter the fetal circulation; the blood levels detected in the fetus are about half that of the mother [33]. The prevalence of major congenital structural malformation in women on calcineurin inhibitors is approximately 4 to 5% and is comparable to the reported incidence in general population of 3 to 4% [4, 34]. Cyclosporine has been shown to increase the production of thromboxane and endothelin increasing vascular bed resistance, which is implicated in the pathogenesis of preeclampsia. It also increases the risk of low birth weight babies, IUGR, and small for gestational age babies. Animal studies have shown that in utero exposure to calcineurin inhibitors causes hypoplastic peripheral lymphatic organs, immature T cells, and nonfunctional T cell reactivity [35]. In human neonates, cyclosporine may cause immature T cell and low number of B cells which may possibly lead to development of autoimmunity [36]. Developmental delays were found in 16% of children with mean age of 4.4 years who received cyclosporine in utero [37]. However, data on pediatric neurocognitive follow-up are limited and the long-term consequence of in utero exposure to calcineurin inhibitors remains limited [38, 39]. There is a 20–25% dose elevation of calcineurin inhibitors of prepregnancy levels required during gestation due to increase in both the volume of distribution and metabolic activity of cytochrome P450 3A pathway. Cyclosporine trough levels decrease by an average of 23% in the first trimester, 39% in the second trimester, and 29% in the third trimester [40]. We recommend more frequent monitoring of the whole blood trough level during pregnancy with biweekly levels in first and second trimesters, weekly levels in third trimester, and repeating levels within a week postpartum.

### 8.2. Azathioprine

Azathioprine is a prodrug that is metabolized rapidly to 6-mercaptopurine and is safe to be used as immunosuppression in pregnancy, even though it has been listed a class D drug by FDA. 6-Mercaptopurine passes into the fetal circulation but fetal liver lacks the enzyme inosinate pyrophosphorylase required for the conversion to active metabolite thioinosinic acid and therefore the fetus is protected from its adverse effect [41]. Azathioprine is teratogenic in rats in high doses of 6 mg/kg of body weight but no anomalies have been described in doses ≤2 mg/kg in the offspring. It is also associated with dose related myelosuppression in fetus but neonatal leukopenia is usually rare, if maternal white cell count is greater than 7500/mm^3^ [42].

### 8.3. Corticosteroids

Commonly used steroids in renal transplant recipients include prednisone (category B) and methylprednisolone (category C) [16, 22, 32]. The placental metabolism of corticosteroids is efficient with 90% of the maternal dose being metabolized in placenta before it reaches fetus; and maternal to cord blood ratio is approximately 10 : 1 [43]. Sporadic cases of fetal adrenal immunosuppression, thymic hypoplasia, and cleft palate have been reported usually at doses more than 20 g/day [44]. In addition, steroids increase the risk of premature rupture of membranes and maternal hypertension during pregnancy. Treatment of allograft rejection with steroids if warranted during pregnancy is not contraindicated.

### 8.4. Mycophenolate Mofetil

Mycophenolate mofetil is a category D drug and is associated with increased risk of spontaneous abortion and congenital malformation. Limb and facial anomalies are the most common congential malformations and include microtia, hypoplastic nails, shortened fifth finger, cleft lip and palate, congenital diaphragmatic hernia, and congenital heart defects [45]. Mycophenolate mofetil is contraindicated in pregnancy and should be stopped 6 weeks prior to conception. It remains unclear as to what to do in the setting of unplanned pregnancy; however, decision should be based on each individual patient after appropriate counseling has been provided. The risk of malformations is not increased in pregnancies fathered by transplant recipients taking mycophenolate [46]. The incidence of congenital anomalies is 23% and miscarriage rate is 49% in babies born to women on mycophenolate as reported in NTPR [4]. Risk evaluation and mitigation strategy (REMS) should be done in all women with childbearing potential and must be on contraception while taking mycophenolate.

### 8.5. Sirolimus

Sirolimus is a category C drug. In animal studies, it has been associated with increased fetal mortality, decreased fetal weights, and delayed ossification of skeletal structure, but no teratogenicity was noted [26]. Data is limited with human exposure but sirolimus is contraindicated in pregnancy and should be stopped 6 weeks prior to conception [22, 47].

## 9. Labor and Delivery

Vaginal delivery is the preferred route of delivery and cesarean section is indicated only for obstetric indications. The renal allograft, which is located in the false pelvis, is not obstructive to the delivery of the fetus. Spontaneous labor can be allowed up to 38 to 40 weeks if there are no obstetrical complications. Stress dose steroids should be given to women during labor who are maintained on steroids for immunosuppression [25]. Pregnancy in renal transplant recipient is high risk and should be managed by a multidisciplinary team of high-risk obstetrician, neonatologist, and transplant nephrologist [22]. We recommend a close follow-up with transplant nephrologist every 2 weeks during the prenatal care.

## 10. Contraception

An unplanned pregnancy after transplantation may expose both mother and fetus to risk of adverse outcomes and puts them at higher risk for induced abortion. More than 90% of the pregnancies were unplanned after transplantation as reported in British transplantation clinic. Among the women who received a transplant, only 48.7% were advised to use contraception and 72.1% were actually using the contraceptive method [12]. It is therefore recommended that women in childbearing age should receive contraceptive counseling as part of their routine care, which should start before transplantation and effective contraceptive method should be started immediately after transplantation. Women who have not been immunized to rubella should receive the vaccination before transplantation, since live viral vaccines are contraindicated after transplantation. Data on the ideal contraceptive method in renal transplant recipients remain limited. Barrier method is not an optimal form of contraception due to potential for contraception failure. Intrauterine devices may increase the chances of infection and in addition lead to contraceptive failure due to reduced anti-inflammatory properties and decreased effectiveness in association with immunosuppressive drugs [48]. The American Society of Transplantation Consensus Conference has suggested the use of low dose estrogen/progesterone or progestin only oral contraceptives in renal transplant recipients if hypertension is well controlled [29]. Center for Disease Control recommends use of either hormonal methods or intrauterine device in uncomplicated solid organ transplant recipients. However, in complicated solid organ transplant patients with acute or chronic allograft failure, combined estrogen/progesterone methods pose unacceptable health risks [49]. Surgical contraception like tubal ligation should be advised in women who have completed their families. Physicians must discuss risk and benefit of each contraceptive method with renal transplant recipients and take into account their desire and lifestyle to determine the most effective contraception.

## 11. Breast-Feeding

Transplant recipients taking prednisone, azathioprine, cyclosporine, and tacrolimus should not be discouraged from breast-feeding [50]. It is well established now that the infants who are breast-fed by mothers on prednisone, azathioprine, and cyclosporine/tacrolimus have a lesser exposure via breast milk than in utero and they do not have adverse effects. The estimated absorption of tacrolimus from breast milk is equivalent to 0.23% of the weight-adjusted maternal dose, which is negligible; and breast-feeding does not slow the decline of tacrolimus levels in infant from higher levels at birth [51]. Infants breast-fed by women on cyclosporine receive less than 300 mcg per day of cyclosporine and absorb undetectable amounts [52]. Exposure of breast milk to corticosteroids is at most 0.1% of total maternal dose and maternal dose of prednisone up to 20 mg/day does not cause adverse effects in infants [53]. Similarly, the amount of azathioprine in breast milk and infant serum is negligible; and breast-feeding is considered safe [54]. Clinical information on breast-feeding is inadequate for mycophenolic acid, sirolimus, everolimus, and belatacept; and breast-feeding should be avoided.

## 12. Conclusion

Renal transplant restores fertility and pregnancy requires careful planning. There should be an expansion of effort by primary care physicians and nephrologists to include the discussion of menstrual and reproductive issues in women with renal transplant. Women of childbearing age wishing to consider pregnancy should receive complete information and counseling from the transplant team. The following summarizes the criteria for renal transplant recipients contemplating pregnancy [29, 32]:

At least 6 months after transplantation
Stable allograft function and creatinine < 1.4 mg/dL
No recent episodes of acute rejection
Blood pressure ≤ 140/90 mmHg
No or minimal proteinuria ≤ 500 mg/24 hours
Prednisone ≤ 15 mg/day
Azathioprine ≤ 2 mg/kg/day
Stopping mycophenolate mofetil and sirolimus 6 weeks prior to conception

Prepregnancy counseling with the potential risks will enable pregnancy planning and help parents make an informed decision. A multidisciplinary approach by the transplant nephrologist and maternal-fetal medicine is essential throughout pregnancy and can result in good outcomes for mother and infant. Due to lack of prospective data, further research is needed in this field which will help us expand our current knowledge.

## Figures and Tables

**Table 1:** Common immunosuppressive drugs use in transplantation.

	FDA pregnancy category
*Induction*	
Basiliximab	B
Alemtuzumab	C
Antithymocyte globulin	C
Methylprednisolone	C
*Maintenance*	
Azathioprine	D
Cyclosporine	C
Tacrolimus	C
Mycophenolate mofetil	D
Sirolimus, rapamycin	C
Prednisone	B
Belatacept	C
Leflunomide	X
*Treatment of rejection*	
Antithymocyte globulin	C
Basiliximab	B
